# Congenital Nasal Pyriform Aperture Stenosis in Association With Polymalformative Syndrome of the Midline

**DOI:** 10.7759/cureus.45153

**Published:** 2023-09-13

**Authors:** Robert Tropsek, Mihaela Horoi, France Ziereisen

**Affiliations:** 1 Radiology, Centre Hospitalier Universitaire (CHU) Saint-Pierre, Bruxelles, BEL; 2 Otolaryngology - Head and Neck Surgery, Centre Hospitalier Universitaire (CHU) Saint-Pierre, Bruxelles, BEL

**Keywords:** panhypopituitarism, single median incisor, pediatric respiratory failure, syndrome of the midline, congenital nasal pyriform aperture stenosis

## Abstract

Congenital nasal pyriform aperture stenosis (CNPAS) is a rare neonatal entity characterized by a reduction in the pyriform orifice of the nasal cavity. Because of its nonspecific clinical presentation as respiratory distress symptoms, it can mimic choanal atresia. Although isolated forms have been described, CNPAS is often associated with other congenital midline malformations. A single median incisor is usually found, with or without other cervical and maxillofacial malformations. The existence of hypothalamic-pituitary axis malformations with endocrine disorders is also possible and, in some cases, a moderate to severe intellectual deficit in association with other brain malformations. Radiological investigation is a central point in the multidisciplinary management of this type of polymalformative syndrome.

## Introduction

Congenital nasal pyriform aperture stenosis (CNPAS) is a rare neonatal entity with an estimated frequency of 1/25,000. It is characterized by a reduction in the pear-shaped pyriform orifice of the nasal cavity [1,2]. According to Brown et al. [1], the narrowness of the orifice is due to excessive growth of the nasal process of the maxilla. A small change in the section area of the pyriform orifice implies significant changes in the airways’ resistance, thus limiting the passage of air. Since newborns are obligate nasal respirators, stenosis of the pyriform orifice leads to episodes of respiratory distress and feeding deficit, especially during breastfeeding. Superficial breathing of high frequency punctuated by cycles of apnea and episodes of cyanosis is typically relieved by the crying of the child [1,2]. This entity is not to be confused with choanal atresia, which is the first cause of nasal congenital abnormality. The latter presents as a complete communication defect between the nasal cavity and the nasopharynx, either unilaterally or bilaterally. Its frequency is estimated at 1/8000 [3,4]. CNPAS is often associated with other congenital malformations, but isolated forms have been described in 20% of cases [5].

## Case presentation

**Figure 1:**
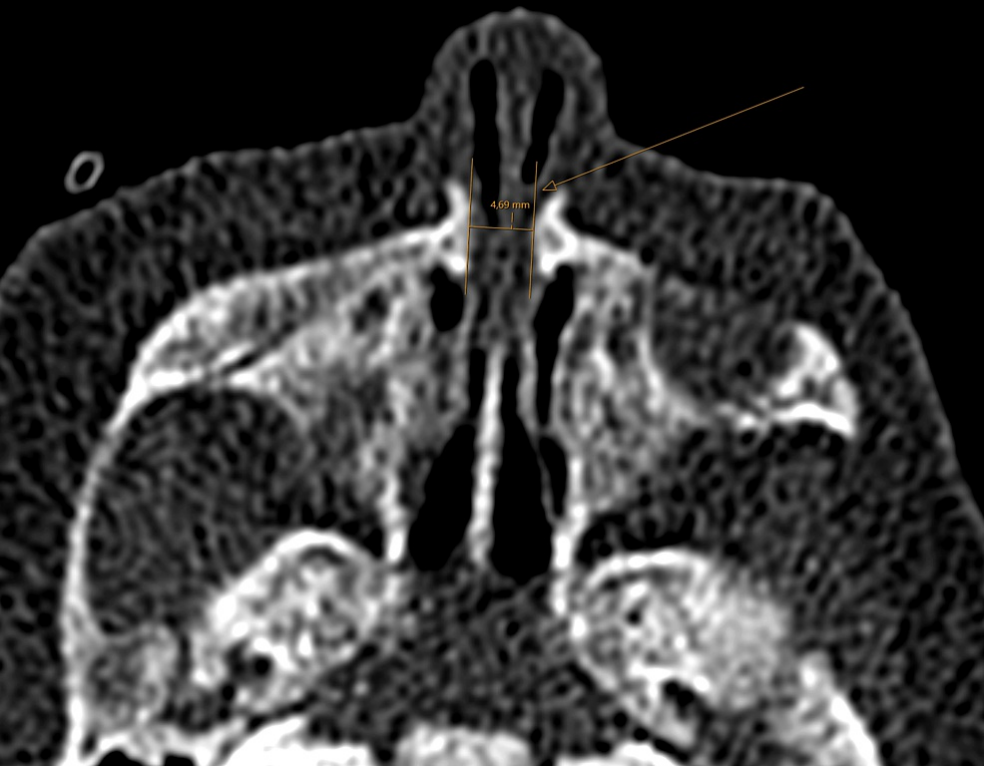
CT scan shows the narrowing of the piriform sinuses in a three-month-old female. The stenosis was 4.7 mm (arrow). CT: computed tomography

**Figure 2:**
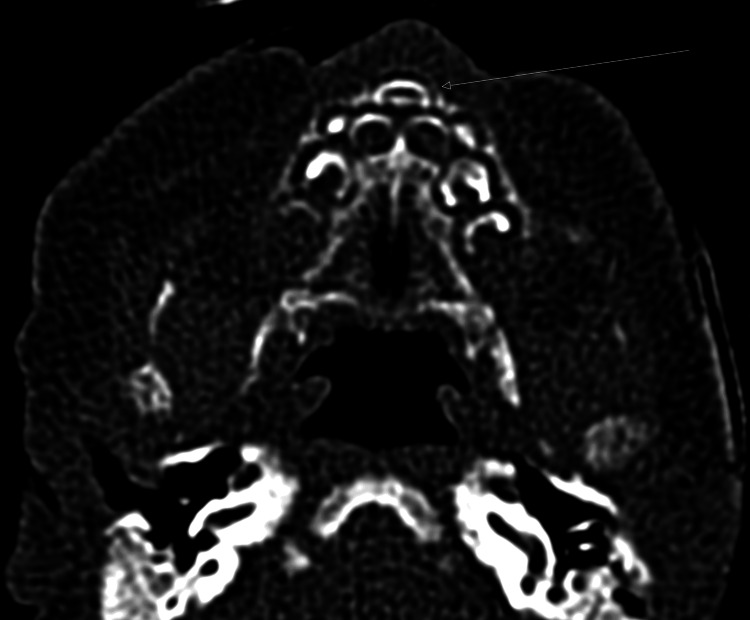
A single median incisor (arrow) in a three-month-old female on axial slices.

A three-month-old female born almost at term vaginally (37 6/7 weeks) without consanguinity presented respiratory distress and cyanosis at birth. The introduction of the nasal tube proved difficult, and the diagnosis of choanal atresia was suspected. A computed tomography (CT) scan was requested and showed the existence of a stenosis of the piriform sinuses (Figure 1). The CNPAS was confirmed as the cause of respiratory distress and obstructive apnea, which required 12 days of nasal high-flow therapy. A nasogastric tube placement was necessary to prevent nutritional deficit. On day 8, under general anesthesia, the stenosis was endoscopically evaluated, and the inferior turbinates were resected and lateralized. The piriform aperture was dilated using Hegar candles up to 4 mm during the same procedure. No stent placement was required. Postoperatively, the patient’s respiratory outcome was favorable. The assessment revealed the existence of a midline syndrome, including a single median incisor (Figure 2) and ante-pituitary insufficiency confirmed by the absence of a pituitary gland on the magnetic resonance imaging (MRI) performed. The patient presented with hypoglycemia and corticotropic and thyroid deficiency, which were subsequently substituted. An abdominal ultrasound revealed no associated malformation. Feeding autonomy in the breast/bottle was acquired from day 23 of life. Currently, the child is gaining weight with a good ponderal index and neurodevelopmental evolution.

## Discussion

The diagnosis of CNPAS is based on endoscopic assessment [3]. A CT scan can confirm and perform a differential diagnosis or look for other malformations. Several authors consider the analysis of the pyriform orifice in the axial section to be abnormal below 11 mm (Figure 2) [3,6-9]. As with other nasal obstructions linked to congenital malformations, CNPAS is classically associated with abnormalities in the development of midline structures and the central nervous system (CNS), which needs to be investigated [3].

Different surgical procedures were described; non-invasive local dilation and piriform aperture drilling with or without local stenting are used, depending on the stenosis diameter and clinical symptoms [10].

Maxillofacial malformations

We find the existence of a single median incisor that constitutes a rare malformation; its frequency is estimated at 1/50000 with a female predominance. The single dental and alveolar canal is present in the primary and definitive dentition [11]. The exact cause of this odontological anomaly is currently unknown. Nevertheless, it is admitted that an in utero malformation event concerning the midline occurring between 35 and 38 weeks would be an issue. Chromosomal depletion of the short arm of chromosome 18p and a mutation of the sonic hedgehog gene on the terminal portion of chromosome 7q have been described by different authors [12,13]. Nearly 90% of children with a single median incisor have a congenital midline malformation [2,13].

A V-shaped palate can be found with an unusual morphology of the mid-palate suture from the palate papilla to the posterior edge of the hard palate [2]. The absence of labial brake and palatal papilla and hypotelorism have been described [13].

Central nervous system (CNS) malformation

Association with CNS abnormalities such as microcephaly or cervical half-vertebrae is not uncommon and should be investigated by MRI. Moderate to severe intellectual deficit may be present, as well as panhypopituitarism in the absence of a pituitary gland [14]. The associated hormonal disorder may occur in a small stature described in 50% of these children [11]. Other abnormalities may be present, such as a single kidney, cardiac malformations, esophageal or duodenal atresia, a micropenis, and genital ambiguity [11].

## Conclusions

CNPAS is a rare entity that can mimic choanal atresia. The primary CT scan assessment must include the search for other cervico-maxillofacial malformations associated with polymalformative midline syndrome. In addition to a hormonal analysis, the complementary assessment should include a brain MRI and abdominal ultrasound.

Management requires a multidisciplinary approach, particularly at the surgical level for the most severe cases, to avoid respiratory disorders and failure to thrive on the endocrine level to replace any deficiency of the hypothalamic-pituitary axis.
